# Graded nomograms based on perioperative parameters for predicting New-Onset severe acute kidney injury following liver transplantation in patients with normal preoperative renal function: the SALT scale

**DOI:** 10.1080/0886022X.2025.2553809

**Published:** 2025-09-10

**Authors:** Chen Chen, Xiaolan Chen, Yuan Gao, Yuxiao Deng, Zhe Li

**Affiliations:** ^a^ Department of Critical Care Medicine, Renji Hospital, School of Medicine, Shanghai Jiao Tong University, China; ^b^ Department of Emergency, Shanghai Pulmonary Hospital, China; ^c^ Department of Liver Surgery, Renji Hospital, School of Medicine, Shanghai Jiao Tong University, China

**Keywords:** Liver transplantation, severe AKI, TEG, nomogram, SALT scale

## Abstract

This study aimed to develop a predictive model and construct a graded nomogram to estimate the risk of severe acute kidney injury (AKI) in patients without preexisting kidney dysfunction undergoing liver transplantation (LT). Patients undergoing LT between January 2022 and June 2023 were prospectively screened. Severe AKI was defined as Kidney Disease: Improving Global Outcomes stage 3. Using the least absolute shrinkage and selection operator (LASSO) analytics, we identified the preoperative, intraoperative, and postoperative factors associated with severe AKI. Machine learning were employed to develop predictive models, and the most suitable model was selected for further analysis. The Shapley Additive Explanation was utilized to construct graded nomograms, forming the Severe AKI post-LT (SALT) scale. Among the 405 patients, 44 had AKI stage 3 (severe AKI). The Model for End-Stage Liver Disease (MELD) score, estimated blood loss, alanine aminotransferase, D-dimer, and thromboelastography reaction time within 24 h post-LT were identified as risk factors. The logistic regression model achieved the highest area under the receiver operating characteristic curve (AUROC) of 0.885. The graded SALT scale, based on the logistic regression model, achieved AUROCs of 0.751, 0.826, and 0.894. The AUROCs for the testing cohort is 0.791. This preliminary study provides a SALT scale for assessing the occurrence of severe AKI after LT. Although additional data are needed to externally validate our model before applying it to patient care, our findings suggest that the SALT scale may be a feasible bedside tool for assessing the risk of AKI after LT.

## Introduction

Liver transplantation (LT) is a lifesaving treatment for patients with end-stage liver disease [1]. Nevertheless, nearly half of liver transplant recipients are at risk of developing acute kidney injury (AKI) postoperatively [2]. Severe AKI (stage 3) necessitates extended renal replacement therapy during hospitalization and prolongs the convalescence period [3–5]. Clinically, severe AKI (stage 3) is diagnosed based on elevated serum creatinine (Scr) levels or oliguria persisting for at least 12 h, though the diagnosis is often delayed. Timely prediction of the occurrence of severe AKI may assist clinicians in ­implementing early interventions [6,7].

A predictive approach incorporating preoperative disease status, intraoperative variables, and postoperative laboratory factors might be suitable for the management of severe AKI (stage 3) after LT. Further development is needed to refine such predictive models.

Machine learning (ML) methods have been employed to predict AKI across various settings [8–10]. A retrospective study demonstrated that ML outperformed traditional regression in predicting post-LT AKI [11]. Building on this, our study aimed to assess the feasibility of ML techniques, including regression, to predict severe AKI (stage 3) after LT in patients without preexisting kidney dysfunction. Furthermore, we aimed to identify a clinically applicable predictive model and developed a graded nomogram, the Severe AKI post-LT (SALT) scale, incorporating parameter weights and hospital feasibility.

## Methods

### Ethical approval

This observational cohort study was conducted at the Department of Critical Care and Hepatology at Renji Hospital, School of Medicine, Shanghai Jiao Tong University. The protocol was approved by the local Ethics Committee (KY2021-019), and written informed consent was obtained from all patients. The study protocol followed the guidelines for the Transparent Reporting of a Multivariable Prediction Model for Individual Prognosis or Diagnosis + Artificial Intelligence [12].

### Study population

Patients admitted to the transplant intensive care unit between January 2022 and June 2023 were included if they met the following criteria: (1) ≥18 years old and (2) undergoing LT. Excluded were patients with preexisting renal dysfunction (estimated glomerular filtration rate <60 mL/min/1.73 m^2^, chronic kidney disease, hepatorenal syndrome, etc), those undergoing combined liver-kidney transplantation, and individuals unwilling to sign an informed consent form. All patients underwent deceased-donor LT (DDLT), without cross-match incompatibility, and followed a local immunosuppressive protocol of basiliximab and delayed tacrolimus.

### Definition of severe AKI

Post-LT severe AKI was defined according to the Kidney Disease: Improving Global Outcomes 2012 guidelines [13], as a three-fold rise in Scr, Scr >354 μmol/L within 48 h, initiation of continuous renal replacement therapy (CRRT), urine output less than 0.3 mL/kg/h for more than 24 h, or anuria persisting for more than 12 h within 7 d post-surgery. The non-severe AKI group included patients without AKI or with AKI stage 1–2.

### Data collection

Demographic data, pre-LT evaluations, non-confidential intraoperative information, and laboratory parameters within 24 h after LT were collected. Urine output per hour was continuously monitored for 7 days, while daily urine output and Scr levels were tracked for 14 days. The requirements for CRRT and the 14-day mortality rates were recorded.

### Statistical analysis

Based on our previous research [14], the study population was calculated assuming a 12% incidence of severe AKI post-LT. Peer-reviewed studies have reported an average of five risk factors associated with AKI following LT [15,16]. Therefore, the sample size was calculated based on an expected event rate and an average of 10 events per variable (EPV) [17,18]. Accounting for a dropout rate of 10%, a sample size of 457 was set. The final cohort included 405 patients with 44 events (EPV = 8.8). However, the training cohort includes only 243 subjects (EPV = 5.2), which is below our target number and may increase the risk of model overfitting. The study employed the least absolute shrinkage and selection operator (LASSO) and cross-validation to ensure robust model development. To partially address this issue, we performed a *post hoc* retrospective analysis of an additional 132 patients—a testing cohort—who underwent LT between January 2024 and June 2024.

Continuous data are presented as mean ± standard deviation (SD) or as median with interquartile range (IQR). Depending on the distribution, comparisons were performed using either the t-test or the Wilcoxon test. Categorical variables were compared using the chi-square or Fisher’s exact tests. Risk factors were identified using LASSO regression. Missing data were imputed using the multiple imputation method in SPSS.The development of the predictive models utilized ML techniques. To guarantee the robustness and generalizability of the models, the patients were randomly assigned to the training and validation cohort in a 6:4 ratio. We employed a multi-model approach, including eXtreme Gradient Boosting (XGBoost), logistic regression, random forest, adaptive boosting (AdaBoost), multilayer perceptron, support vector machine, and K-nearest neighbors, and the model with the highest average area under the receiver operating characteristic (AUROC) curves was prioritized for optimal selection. The models were implemented in Python (version 3.7) using Scikit-learn 0.22.1 and XGBoost 1.2.1. The model performance and generalizability were ensured through a 10-fold cross-validation with grid search. Model performance was evaluated through ROC curves, decision curve analysis (DCA), and precision-recall (PR) curves. The Shapley Additive Explanations (SHAP) representation analysis determined factor significance, aiding the development of graded nomograms (SALT Scale). Data analyses were conducted using the R software (version 4.1.2) and Python. Statistical significance was defined as *p* < 0.05.

## Results

### Perioperative characteristics and severe AKI occurrence

**Figure 1.**
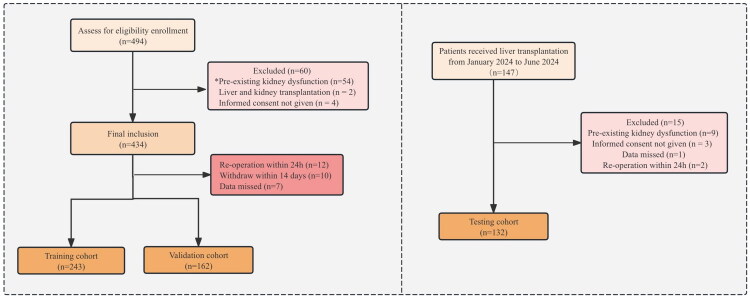
Flowchart of the study process. *Preexisting kidney dysfunction (estimated glomerular filtration rate <60 mL/min/1.73 m^2^).

During the study period, 474 adults received LT, of which 60 met the exclusion criteria. Data from 29 patients were excluded due to reoperation within 24 d, withdrawal within 14 d, and missing data. Consequently, 405 patients were deemed eligible for model development (Figure 1). Forty-four patients developed postoperative severe AKI (stage 3), with the average diagnosis time being 27.1 ± 4.4 h. This group demonstrated elevated 14-d mortality rates and an increased demand for CRRT. Additionally, both groups had similar baseline Scr levels. However, the severe AKI (stage 3) group had a higher prevalence of acute liver failure and elevated Model for End-Stage Liver Disease (MELD) scores.

Intraoperatively, the severe AKI (stage 3) group experienced, on average, 400 mL more blood loss and required greater volumes of red blood cells and plasma transfusions. Within 24 h postoperatively, the severe AKI (stage 3) group exhibited impaired coagulation function, reflected by the D-dimer and thromboelastography reaction time (TEG-R) values (Table 1).

**Table 1.** Demographic and clinical characteristics of the study population.

Subjects	Overall *n* = 405	Non Severe AKI *n* = 361	Severe AKI *n* = 44	P value
Demographic data				
Male (*n*, %)	295 (72.8)	258 (71.7)	37 (84.1)	0.116
Age [mean ± SD, y]	50.2 ± 10.9	50.0 ± 11.1	51.9 ± 9.9	0.269
Comorbidities and etiology of liver transplantation				
Diabetes mellitus (*n*, %)	62 (15.3)	56 (15.6)	6 (13.6)	0.899
Hypertension (*n*, %)	33 (8.1)	30 (8.4)	3 (6.8)	0.609
Cirrhosis				
Hepatitis B (*n*, %)	133 (32.8)	118 (33)	15 (34.1)	1.000
PBC (*n*, %)	28 (6.9)	27 (7.5)	1 (2.3)	0.342
Alcoholic (*n*, %)	17 (4.2)	15 (4.2)	2 (4.5)	1.000
AIH (*n*, %)	25 (6.2)	22 (6.1)	3 (6.8)	0.746
Other (*n*, %)	30 (7.4)	26 (7.3)	4 (9.1)	0.556
Liver cancer (*n*, %)	150 (37.0)	136 (38.0)	14 (31.8)	0.526
Acute hepatic failure (*n*, %)	140 (34.6)	115 (32.1)	25 (56.8)	0.002
Other (*n*, %)	9 (2.2)	9 (2.5)	0 (0)	0.606
Ascites [M(*P*_25_, *P*_75_), mL]	200 (0–1600)	200 (0–1500)	400 (0–200)	0.315
Meld score [M (*P*_25_, *P*_75_)]	13.0 (8.52–19.0)	12.0 (8.0–17.9)	16.6 (11.2–31.4)	<0.001
Baseline Scr [M(P25, P75), μmol/L]	67.0 (54.0–81.0)	67.0 (55.0–81.0)	62.0 (50.0–81.0)	0.417
Operation details				
EBL [M(*P*_25_, *P*_75_), mL]	600 (400–1000)	600 (400–800)	1000 (600–1500)	<0.001
RBC transfusions [M (P_25_, P_75_), Units]	4.0 (0–8.0)	4 (0–8)	8 (4–10.89)	<0.001
Plasma transfusions [M (P_25_, P_75_), mL]	500 (0–1000)	400.0 (0.0–1000.0)	950.0 (400.0–1450.0)	<0.001
Anhepatic period [M (P_25_, P_75_), min]	35.0 (35.0–35.0)	35.0 (35.0–35.0)	35.0 (35.0- 35.0)	0.752
Warm ischemia time [M (P_25_, P_75_), min]	13.6 ± 2.1	13.5 ± 2.1	14 ± 2.3	0.141
Cold ischemia time [M (P_25_, P_75_), min]	6.7 ± 0.7	6.7 ± 0.7	6.8 ± 0.6	0.365
Postoperative laboratory variables within 24h				
ALT [M(*P*_25_,*P*_75_), μ/L]	529.0 (279.0- 959.0)	500.0 (273.0–912.0)	865.5 (398.0–2048.5)	0.002
AST [M(P_25_,P_75_), μ/L]	963.0 (463.0–2165.0)	887.0 (439.0–1992.0)	2496.0 (817.0–7305.0)	<0.001
HB [M(P_25_, P_75_), g/L	78.0 (67.0–90.0)	79.0 (68.0–92.0)	69.5 (61.0–79.5)	<0.001
PLT [M(P_25_,P_75_), 10^9^/L]	50.0 (31.0–81.0)	53.0 (32.0–84.0)	37.0 (23.0–58.5)	0.004
INR [M(P_25_,P_75_)]	1.57 (1.34–1.90)	1.5 (1.3–1.9)	1.9 (1.5–2.4)	<0.001
D-dimer[M(P_25_,P_75_), μg/mL]	2.18 (1.09–4.52)	2.0 (1.0–3.7)	5.7 (2.4–31.7)	<0.001
FDP [M(P_25_,P_75_), μg/mL]	14.29 (8.77–25.9)	13.2 (8.3–23.9)	31.0 (15.8–118.4)	<0.001
TEG index within 24h post LT				
R value [M(P_25_,P_75_), min]	7.2 (6.0–8.8)	7.0 (5.8–8.4)	10.2 (8.3–15.5)	<0.001
K value [M(P_25_,P_75_), min]	4.4 (2.7–7.1)	55.8 (45.0- 64.7)	46.2 (36.9–54.6)	<0.001
α angle [M(P_25_,P_75_), deg]	54.2 (44.0–63.4)	4.2 (2.6–6.5)	6.7 (4.7–10.5)	<0.001
MA [M(P_25_,P_75_), mm]	40.2 (33.0–48.8)	41.1 (33.9–49.9)	34.6 (29.1–40.7)	<0.001
Postoperative outcomes				
Diagnosis time [M(P_25_,P_75_), days]	/	/	1(1,3)	/
CRRT (n, %)	16 (3.9)	2 (0.6)	14 (31.8)	<0.001
Hospital mortality (n, %)	32 (7.9)	15 (4.2)	17 (38.6)	<0.001

AKI: acute kidney injury; MELD: model for end-stage liver disease; RBC: red blood cell: 1 *u* = 200 mL; ALT: alanine transaminase;EBL: Estimated blood loss;CRRT: Continuous renal replacement therapy: PBC: Primary Biliary Cirrhosis; AIH: autoimmune hepatitis; AST: aspartate transaminase; INR: international normalized ratio; FDP: Fibrinogen Degradation Products; HB: Hemoglobin; PLT: platelet; MA: maximum amplitude. Ascites represent amount of abdominal fluid drained during surgery; TEG-R: thromboelastography reaction time.

### Development and comparison of predictive models

**Figure 2.**
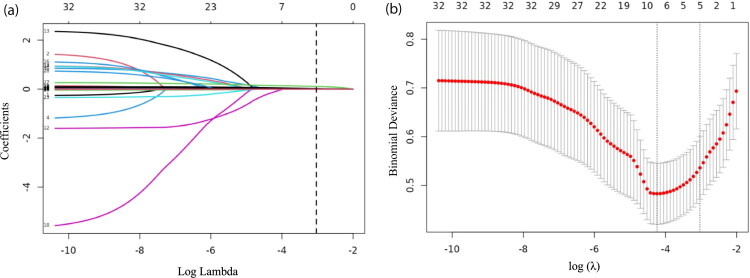
LASSO regression of perioperative risk factors for severe AKI post-LT. (a) The plot of LASSO coefficient profiles. (b) Tuning parameters (λ) selection cross-validation error curve. **Panel (a) tracks how predictor coefficients shrink toward zero as the penalty strength (log λ) increases, automatically filtering out less important variables. Panel (b) reveals a U-shaped curve where prediction error (binomial deviance) initially falls, then rises. The vertical dashed line indicates the optimal log λ value that minimizes error while striking a balance between model simplicity and accuracy. Together, these plots demonstrate variable selection and tuning for an optimal predictive model. LASSO: least absolute shrinkage and selection operator; AKI: acute kidney injury.

A schematic of the nomogram development and selection is illustrated in Figure 1. Through LASSO regression analysis, we identified significant parameters that distinguished the severe AKI group from the non-severe AKI group (non-AKI and stage 1–2) (Figure 2). The five variables with the highest odds ratios were selected for model development: MELD score, intraoperative estimated blood loss, postoperative alanine aminotransferase (ALT), D-dimer, and TEG-R values.

**Figure 3.**
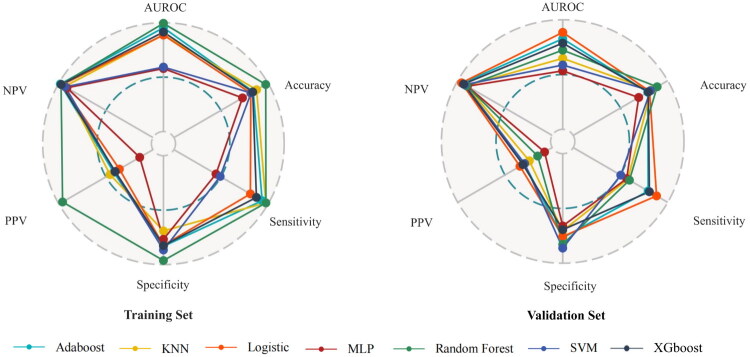
Performance comparison of predictive models for severe AKI post-liver transplantation. **The labels surrounding the figure represent different model evaluation parameters. The center point, along with the colored dashed lines and gray dashed lines, indicates 0%, 50%, and 100% respectively. The colored lines represent different machine learning models, while the corresponding colored dots indicate their performance values for each parameter. AKI: acute kidney injury.

**Figure 4.**
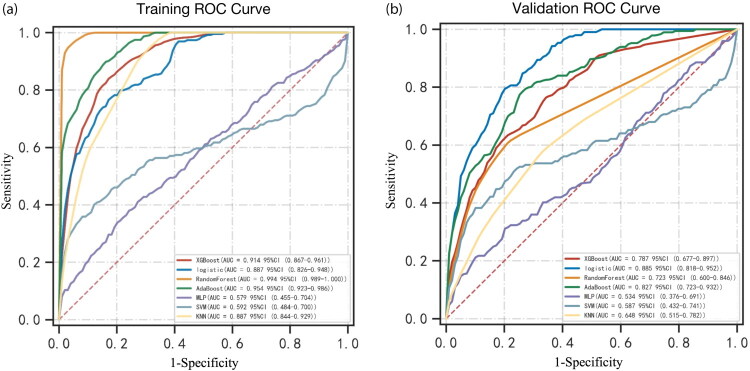
ROC curve of seven machine learning models for predicting severe AKI post-LT. (a) ROC curve of the training cohort. (b) ROC curve of validation cohort. ROC: receiver operating characteristic curve; AKI: acute kidney injury.

**Figure 5.**
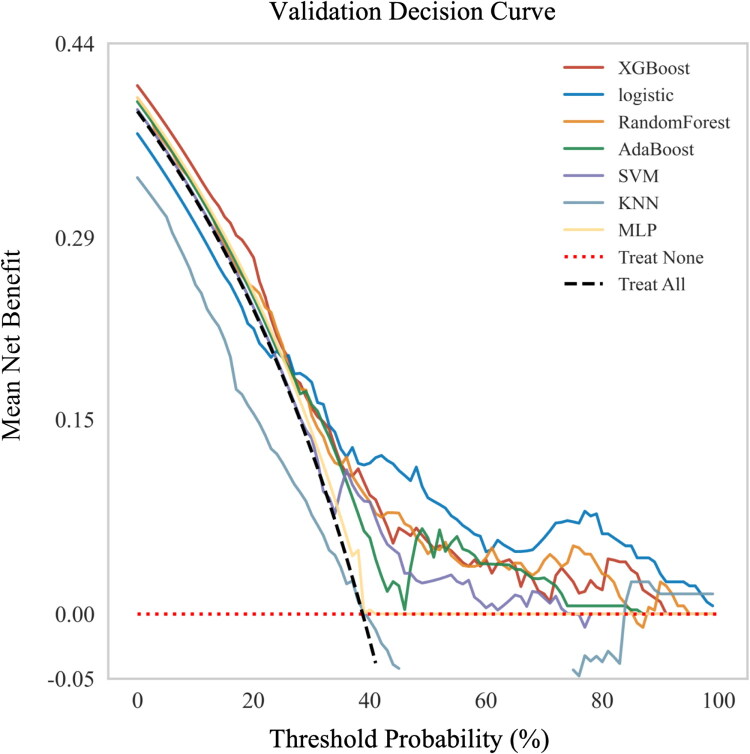
DCA of seven machine learning models for predicting severe AKI post-LT.**This validation decision curve plot compares the clinical utility of multiple predictive models (XGBoost, logistic regression, Random Forest, AdaBoost, SVM, MLP, KNN) using the mean net benefit (y-axis) across varying threshold probabilities (x-axis). ** the curves illustrate how the clinical value of each model varies with different intervention thresholds. Solid-colored lines represent each machine learning model, while dashed lines indicate the ‘treat none’ (no intervention) and ‘treat all’ (universal intervention) strategies. Higher net benefit values indicate better clinical usefulness for decision-making purposes. DCA: Decision curve analysis; LT: liver transplantation; XGBoos: eXtreme Gradient Boosting; AdBoost: Adaptive Boosting; MLP: multilayer perceptron; SVM: support vector machine; KNN: K-Nearest-Neighbor.

Patient data were divided into training and test datasets to develop and validate the predictive models. The 405 patients were divided into training and validation groups at a 6:4 ratio, with the training group consisting of 243 patients (including 26 with severe AKI) and the validation group comprising 162 patients (including 18 with severe AKI). Among the seven ML models, the logistic regression model exhibited the highest AUROC value (0.885) in the validation set. Additionally, the model also demonstrated the highest positive (PPV) (0.357) and negative (0.969) predictive values in both the training and validation cohort (Figures 3 and 4, and Table S1). The logistic regression model also revealed the highest area under the PR curve and the highest net benefit, particularly at a threshold of approximately 75%, as indicated by the DCA (Figure 5). The calibration curves showed that the logistic regression model had the lowest proportion of positive values (Fig. S2). Furthermore, the logistic regression model exhibited only a 10% AUROC difference between the validation and training cohort (Fig. S3). Model configurations and hyperparameters are detailed in Table S2.

### Developing graded nomograms for predicting severe AKI risk post-LT: the SALT scale

**Figure 6.**
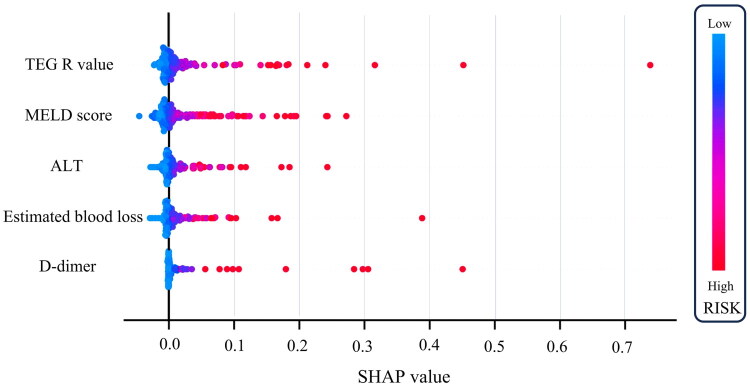
SHAP of the logistic regression model. The SHAP values explain the contribution of each risk factor to the overall outcome. The dots, ranging from blue to red, represent low to high risk values for severe AKI post-LT. SHAP: Shapley Additive Explanations.

SHAP representation revealed that an increased risk of developing severe AKI (stage 3) was associated with elevated MELD scores, greater estimated blood loss volume, elevated ALT levels, increased TEG-R values, and elevated D-dimer levels, with TEG-R having the most significant impact on model output (Figure 6).

**Figure 7.**
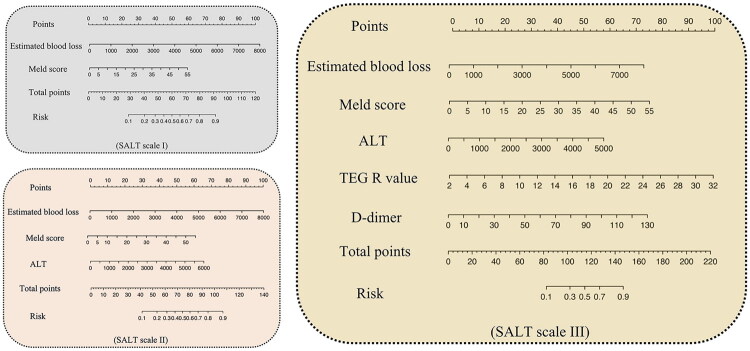
The graded nomograms in the predicting severe AKI post-LT (SALT) scale were developed using the risk factors identified by the logistic regression model. To predict severe AKI risk: Locate each patient variable (EBL, MELD, ALT, TEG R, D-dimer) on its axis to get points, sum all points to find total points, then align total points vertically with the risk axis to read the predicted probability. EBL: estimated blood loss; MELD: model for end-stage liver disease; ALT: alanine transaminase; TEG-R: thromboelastography reaction time.

Based on these findings, the post-LT severe AKI (stage 3) prediction scale (SALT) was developed. The scale consisted of three nomograms, SALT I, II, and III, utilizing either partial or complete variables from the logistic regression model (Table 2). The SALT scale was tailored according to the weights of the parameters, allowing its application across local hospitals with varying laboratory testing capabilities. SALT I, which included the preoperative MELD score and intraoperative estimated blood loss volume, achieved AUROC values > 0.75. SALT II, which incorporated postoperative ALT, improved the diagnostic performance by 6.5% in the net reclassification index compared to SALT I. SALT III, which integrated the TEG-R value and D-dimer level, demonstrated a 27.9% enhancement in diagnostic performance compared to that noted with SALT I. Detailed nomograms for the SALT scale are presented in Figure 7 and their calibration curves in the training and validation cohorts are displayed in Fig. S4.

**Table 2.** Comparison of hierarchical models for early predicting severe AKI post-LT.

	Variables	Univariable		Compared with SALT I	
		AUROC	95% CI	*P* value	NRI
SALT I	MELD score, Estimated blood loss	0.751	0.706–0.793	/	/
SALT II	MELD score, Estimated blood loss, ALT	0.826	0.785–0.861	0.017	0.065
SALT III	MELD score, Estimated blood loss, ALT, D-dimer, TEG-R	0.894	0.860–0.922	0.001	0.279

EBL: Estimated blood loss; MELD: model for end-stage liver disease; ALT: alanine transaminase; TEG-R: thromboelastography reaction time.

### Additional *post hoc* validation of model (testing cohort)

**Figure 8.**
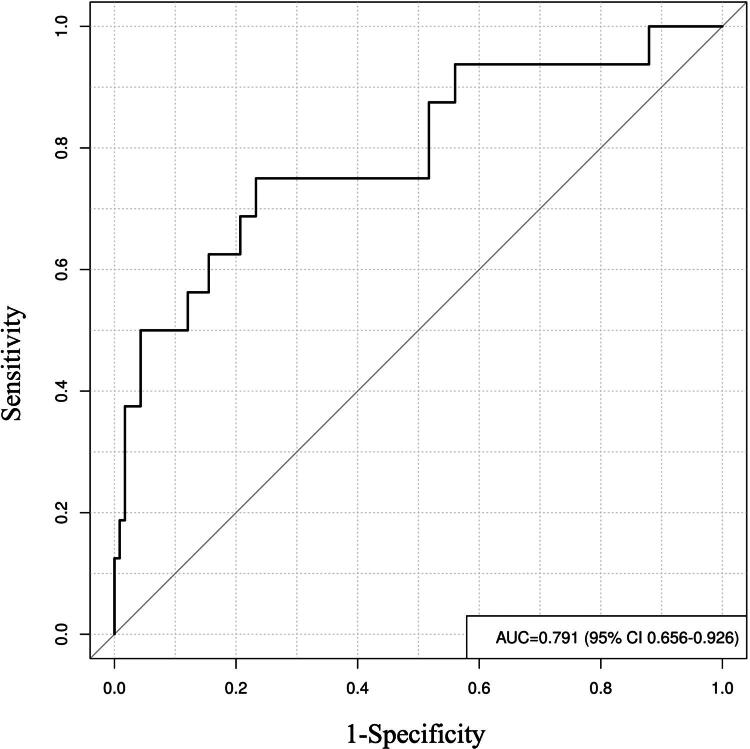
ROC curve of testing cohort. ROC: receiver operating characteristic curve.

**Figure 9.**
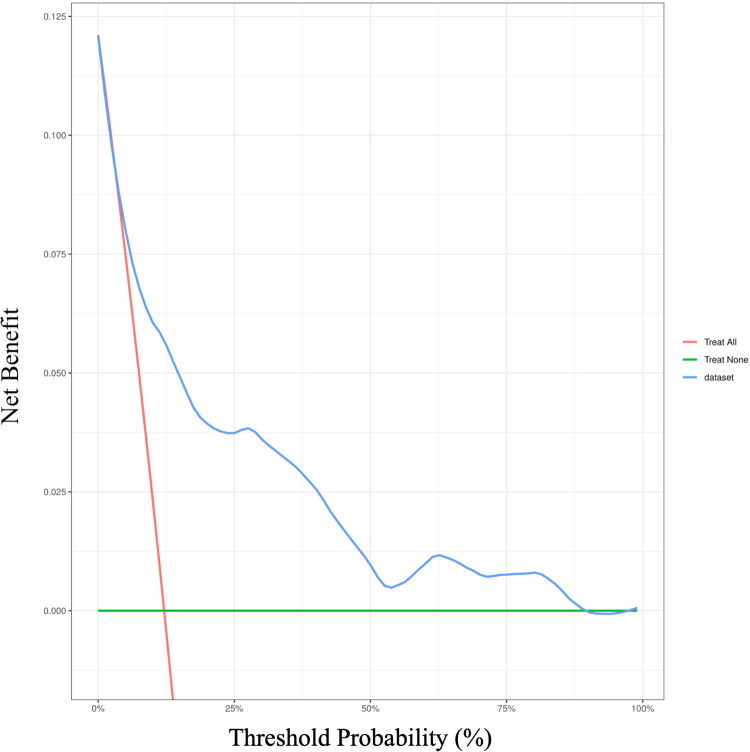
DCA curve of testing cohort. DCA: decision curve analysis.

To provide further internal validation of our model, we retrospectively analyzed data from another 132 LT patients treated between January 2024 and June 2024 as a testing cohort independent of the original cohort. Table S3 presents the specific data of the 132 patients included in the model variables, which yielded an AUC of 0.791 (Figure 8). Additionally, the DCA curve demonstrated the robust clinical utility of our model (Figure 9).

## Discussion

This study created a graded nomogram to predict early severe AKI (stage 3) in patients who underwent LT without preexisting kidney dysfunction. A logistic regression model incorporating MELD score, estimated blood loss, ALT, TEG-R value, and D-dimer levels within 24 h post-LT demonstrated the best performance in our prospective cohort.

AKI following LT is a complex issue driven by multiple factors during the perioperative period [19,20]. In our study, 10.8% of patients developed severe AKI, consistent with rates reported by other centers, ranging from 7% to 16% [21,22]. MELD scores, Scr levels before transplantation, intraoperative estimated blood loss volume, blood product administration, and vasoactive drug utilization during surgery have been identified as risk factors for AKI in general LT recipients [6,19,23]. We previously reported that immediate postoperative coagulation deficiency, which reflects the combined effect of surgery and donor factors on endothelial function, is a significant predictor of severe AKI (stage 3) [14]. We identified substantial differences in MELD scores, intraoperative estimated blood loss, and transfusion between patients with and without severe AKI. This confirms findings of the previous research demonstrating that MELD score and intraoperative estimated blood loss volume influence AKI occurrence [24]. Interestingly, our findings suggest that immediate postoperative coagulation tests, particularly TEG-R and D-dimer levels, exhibit substantial predictive value.

Coagulation function after LT is complex, influenced by end-stage liver disease, intraoperative estimated blood loss, and blood transfusions [25,26]. In our study population, no differences were noted in the clotting times or surgical and immunosuppressive regimens between patients with and without AKI. We suspect that the differences in coagulation function at 24 h post-LT reflect the recipient’s endothelial response to the new liver and intraoperative conditions. Patients with acute liver failure and stage 3 AKI exhibited increased factor VIII and von Willebrand factor (vWF) levels, indicating endothelial activation [27]. Elevated D-dimer levels serve as markers of both endothelial damage and AKI [28–31]. These findings support the role of endothelial cells as key contributors to AKI progression post-LT, being early targets of hypotension, ischemia, and inflammatory responses [32]. Our previous study discovered that postoperative coagulation function within 24 h post-LT serves as an early marker of endothelial injury, predicting severe AKI (stage 3) [14]. Our finding that prolonged TEG-R time (indicating hypocoagulability) predicts severe AKI reveals a unique coagulopathy-endothelial axis in liver transplantation, addressing the paradox where endothelial damage usually promotes hypercoagulability [33]. Rather than initiating prothrombotic cascades, post-LT endothelial disruption creates a biphasic insult: initial reperfusion-mediated coagulation factor consumption [34] combined with the heparin-like effects of shed glycocalyx fragments to overwhelm prothrombotic stimuli such as vWF release. This culminates in NETosis-driven anticoagulation, where neutrophil extracellular traps sequester clotting components, subsequently impairing renal microvascular perfusion by disrupting thrombomodulin-protein C signaling [27,35]. Our data demonstrated that factor depletion exceeded thrombotic potential in LT physiology, highlighting the need for prospective studies on targeted interventions such as recombinant factors or NET inhibitors to disrupt this newly characterized AKI pathway. However, these are merely our inferences, and further research is necessary to understand the pathophysiological mechanisms of endothelial damage after LT and its impact on TEG-R values.

A comprehensive evaluation approach can accurately identify patients at high risk. Berkowitz et al. analyzed the preoperative and postoperative risk factors for AKI following LT [36]. We employed seven ML approaches to explore the complex relationships between perioperative risk factors, including postoperative laboratory tests. The XGBoost, logistic, random forest, and Adaboost models achieved an AUROC of over 0.7 in both the training and validation cohort. Notably, the logistic regression model demonstrated the highest AUROC (0.89 and outperformed the other models in terms of DCA and PR curves in the validation cohort (Figure 3, Fig. S2). Conversely, a previous retrospective study identified that ML outperformed regression in predicting post-LT AKI [11]. This study suggests that the SALT scale warrants validation in additional liver transplant populations and explorations in other primary surgery cohorts. The logistic regression model’s PPV of 35.7% indicated that nearly two-thirds of patients flagged as ‘high-risk’ may not develop severe AKI (false positives). In clinical practice, medical management should be adjusted based on changes in 48-h creatinine levels and novel AKI biomarkers. This study may help identify some modifiable factors in the AKI stage 3 model, guiding targeted interventions.

In our study, 31.8% of patients with severe AKI (stage 3) required CRRT to maintain acid-base and fluid balance, allowing time for renal recovery [2]. However, traditional severe AKI (stage 3) diagnosis, occurring within 12 to 48 h, poses challenges to the timely initiation of treatment [37]. Considering the resource heterogeneity and imbalance, we employed graded-score nomograms (SALT scale) to achieve bedside generalizability. All nomograms, based on indicators obtained within 24 h after LT, demonstrated an AUROC over 0.7, indicating the potential for timely assessment of severe AKI (stage 3).

Our study had several limitations. First, our findings remain preliminary. The observational single-center design and relatively small sample size may limit the generalizability of our findings and increase the risk of overfitting, particularly given the complex interactions between covariates in our model. While we performed internal validation through cohort splitting, the absence of a external validation cohort restricts our ability to confirm the model’s performance in diverse clinical settings. Clinicians should be cautious when using this model. Second, due to recipient privacy policies, obtaining detailed donor liver data and an external validation cohort was challenging. This study also lacks data on confounding factors, such as fluid balance and vasoactive medication use; thus, well-designed multicenter prospective studies are needed in the future. Future multicenter validation should prioritize standardized collection of donor-specific variables (e.g., macrosteatosis percentage, ischemia times), recipient immunosuppressive regimens (e.g., tacrolimus trough levels, glucocorticoid protocols), and center-specific practice patterns (e.g., case volume, anhepatic time management) to refine the SALT scale’s generalizability. As a single-center observational study, the results of our study should be considered hypothesis-generating, and additional external validation studies are required before our findings can be applied to patient care.

## Conclusion

The SALT scale, based on a logistic regression model derived from perioperative factors, demonstrates potential clinical utility for AKI risk stratification in liver transplantation; however, its implementation requires careful consideration of sample size constraints and external validation gaps. Future multi-center studies with larger cohorts are essential to verify its robustness across diverse populations before clinical deployment.

## Supplementary Material

supplement_Table - Clean.docx

Supplementary_Materials- Clean.docx

## Data Availability

The data in this study are available from the corresponding author.
