# Supplementary material for: Graded nomograms based on perioperative parameters for predicting New-Onset severe acute kidney injury following liver transplantation in patients with normal preoperative renal function: the SALT scale
Source: Ren Fail. 2025 Sep 10;47(1):2553809. doi: 10.1080/0886022X.2025.2553809 (PMC12424153; doi:10.1080/0886022X.2025.2553809)
Supplement: supplement_Table - Clean.docx [file IRNF_A_2553809_SM2622.docx]

Table S1. Performance Comparison of Predictive Models for Severe AKI Post-LT

| Model |  | Training set | | | | | |  | Validation set | | | | | |
| --- | --- | --- | --- | --- | --- | --- | --- | --- | --- | --- | --- | --- | --- | --- |
|  |  | AU  ROC | Accu  racy | Sensi  tivity | Speci  ficity | PPV | NPV |  | AU  ROC | Accu  racy | Sensi  tivity | Speci  ficity | PPV | NPV |
| XGboost |  | 0.914 | 0.838 | 0.877 | 0.826 | 0.401 | 0.975 |  | 0.787 | 0.794 | 0.806 | 0.694 | 0.310 | 0.938 |
| Logistic |  | 0.887 | 0.817 | 0.813 | 0.823 | 0.357 | 0.969 |  | 0.885 | 0.808 | 0.882 | 0.756 | 0.341 | 0.960 |
| Random Forest |  | 0.994 | 0.976 | 0.978 | 0.963 | 0.961 | 0.977 |  | 0.723 | 0.888 | 0.595 | 0.828 | 0.154 | 0.905 |
| Adaboost |  | 0.954 | 0.844 | 0.938 | 0.832 | 0.417 | 0.985 |  | 0.827 | 0.789 | 0.793 | 0.768 | 0.294 | 0.952 |
| MLP |  | 0.579 | 0.729 | 0.447 | 0.768 | 0.141 | 0.915 |  | 0.534 | 0.693 | 0.566 | 0.660 | 0.081 | 0.898 |
| SVM |  | 0.592 | 0.820 | 0.492 | 0.866 | 0.405 | 0.932 |  | 0.587 | 0.818 | 0.503 | 0.862 | 0.292 | 0.927 |
| KNN |  | 0.887 | 0.879 | 0.977 | 0.691 | 0.457 | 0.939 |  | 0.648 | 0.826 | 0.578 | 0.704 | 0.242 | 0.908 |

AUROC=area under the receiver operating curve; PPV=Positive predictive value;NPV=negative predictive value; XGBoos=eXtreme Gradient Boosting ; AdBoost=Adaptive Boosting; MLP= Multilayer Perceptron ; SVM=Support Vector Machine;KNN=K-Nearest-Neighbor

Table S2.The tuned hyperparameters of employed machine learning models

| Models | Major parameters |
| --- | --- |
| XGboost | L2 regularization with weight =1  learning rate =0.001  the maximum tree depth =3 |
| Logistic Regression | L2 regularization with weight =1  Maximum Number Of Iterations=100  tolerance=0.0001 |
| RandomForest | criterion= gini, maximum tree depth= 10  minimum impurity decrease= 0.0  number of estimators=20 |
| AdaBoost | learning rate= 1.0  number of estimators=50 |
| MLP | hidden layer sizes= (30, 30)  max number of iterations= 10 |
| SVM | kernel type=rbf  tolerance=0.001 |
| KNN | number of neighbors= 5  weights type= uniform |

XGBoos= eXtreme Gradient Boosting; AdBoost=Adaptive Boosting; MLP=Multilayer Perceptron ;SVM=Support Vector Machine;KNN=K-Nearest-Neighbors

Table S3.Clinical characteristics of the post hoc internal training cohort(testing cohort)

| Subjects | Overall  n=132 | Non Severe AKI  n=116 | Severe AKI  n=16 | P value |
| --- | --- | --- | --- | --- |
| EBL[M(P25,P75),mL] | 800.0(400.0-1000.0) | 600.0 (400.0-1000.0) | 1000.0 (750.0-2000.0) | 0.007 |
| Meld score [M (P25, P75)] | 13.0(8.5;21.9) | 13.0(8.5-19.9) | 16.6 (10.0-33.4) | 0.024 |
| ALT[M(P25,P75),u/l] | 625.50(347.5-1097.2) | 592.50(350.5-1075.0) | 846.0(296.2-1452.2) | 0.458 |
| R value [M(P25,P75),,min] | 7.40(6.2-9.9) | 7.2 (6.2-9.3) | 9.0(8.2;13.9) | 0.008 |
| D-dimer[M(P25,P75), ug/ml] | 2.3(1.2-5.5) | 2.3 (1.1-4.5) | 4.3(2.4-31.8) | 0.006 |

EBL,Estimated blood loss;MELD, model for end-stage liver disease;ALT, alanine transaminase;TEG-R,thromboelastography reaction time
