# Supplementary material for: Graded nomograms based on perioperative parameters for predicting New-Onset severe acute kidney injury following liver transplantation in patients with normal preoperative renal function: the SALT scale
Source: Ren Fail. 2025 Sep 10;47(1):2553809. doi: 10.1080/0886022X.2025.2553809 (PMC12424153; doi:10.1080/0886022X.2025.2553809)
Supplement: Supplementary_Materials- Clean.docx [file IRNF_A_2553809_SM2621.docx]

Supplementary Materials:

# Supplementary Figures:


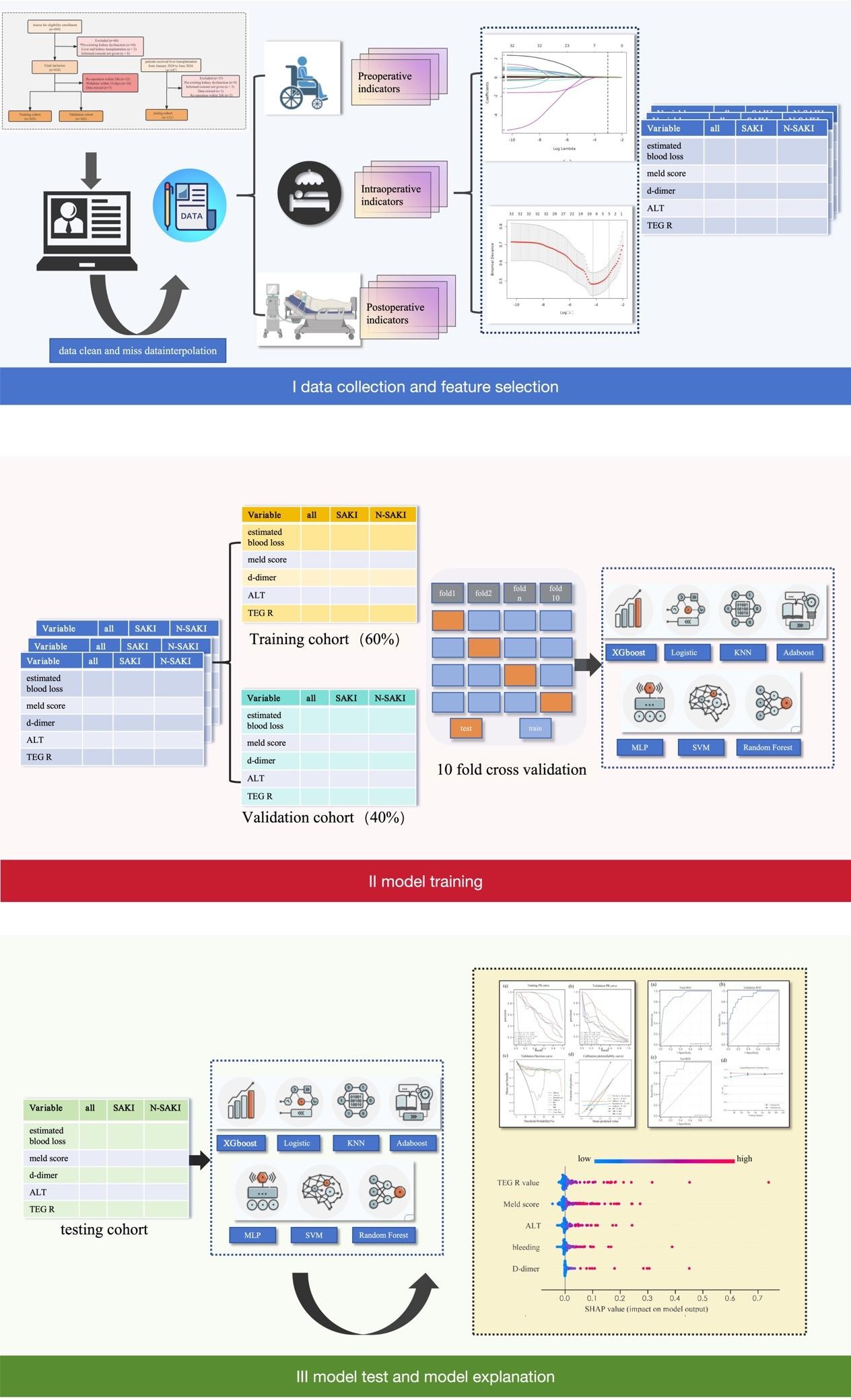


## Fig.S1Schematic diagram of the four-step process for developing nomograms predicting severe AKI post-liver transplantation (SALT Scale). The process begins with data collection and feature selection, where preoperative, intraoperative, and postoperative patient indicators are gathered and features are extracted using Lasso regression. Next, the model is trained and iteratively refined to enhance performance. The trained model is then tested with test data to evaluate its performance, resulting in a confusion matrix and various performance metrics such as AUROC, DCA, and PR curves.AKI,acute kidney injury;AUROC,area under the receiver operating characteristic curve;DCA,Decision curve analysis;PR,Precision-Recall Curve


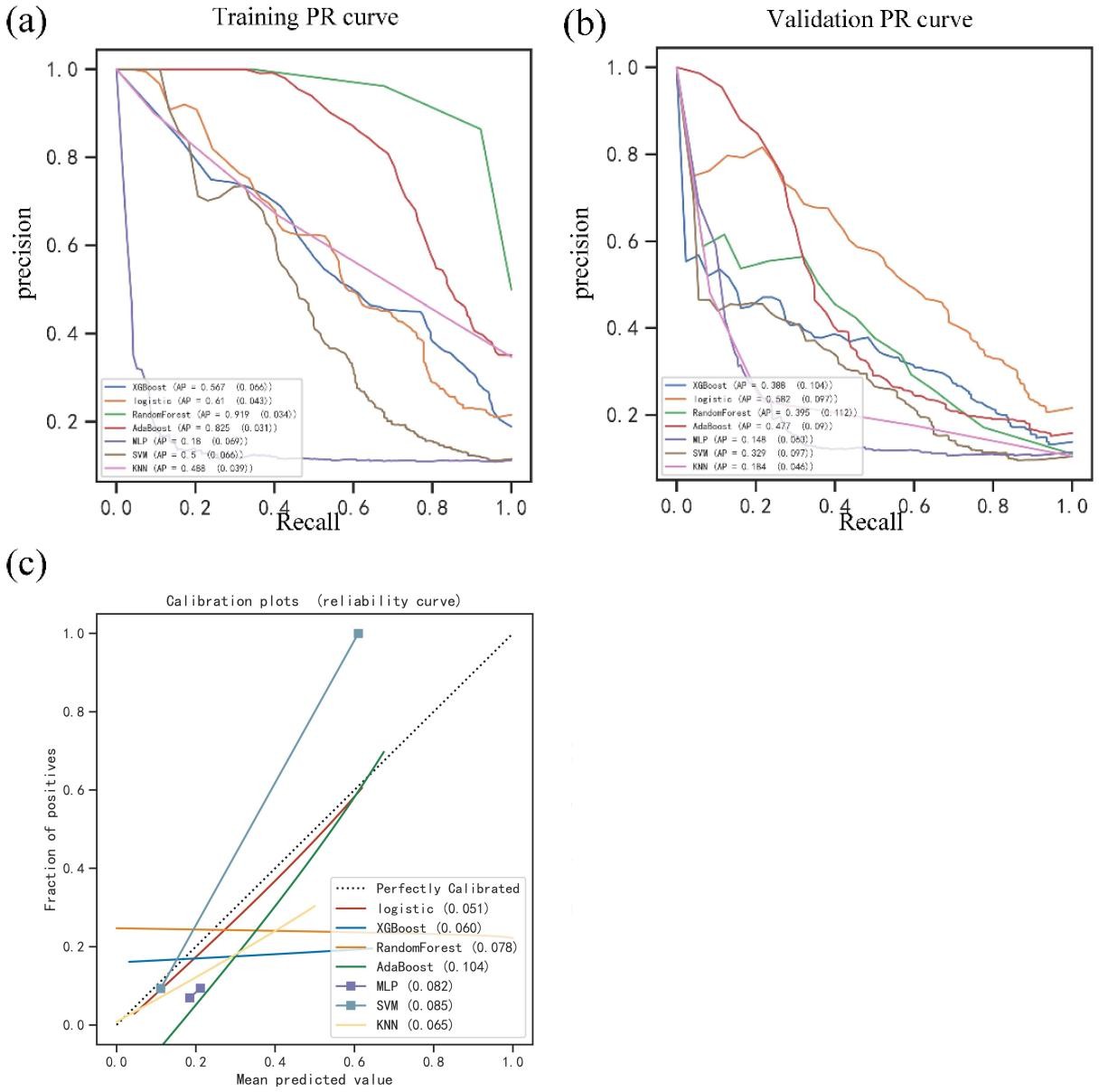


Fig.S2.PR curve and calibration curve of seven machine learning models for predicting severe AKI post-LT. (a) PR curve of the training cohort. (b) PR curve of validation cohort. (c) calibration curve of validation cohort.PR,Precision-Recall Curve;AKI,acute kidney injury


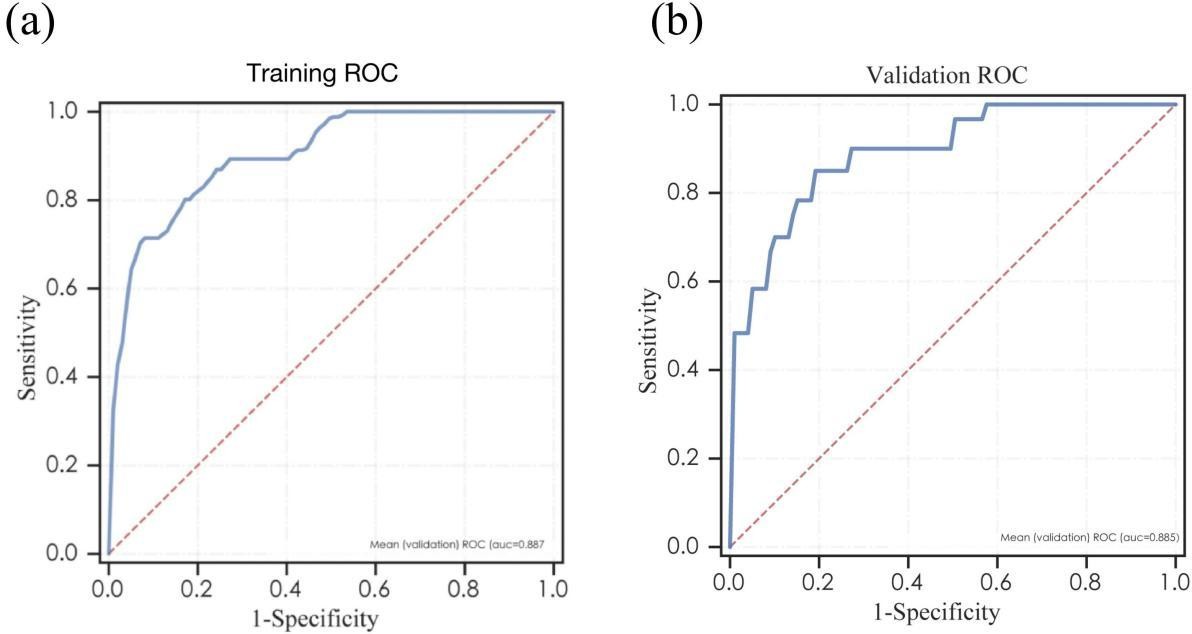


Fig.S3.AUROC curve and learning curve of the logistic regression model. (a)ROC curve for the

training (b)ROC curve for the validation .

AUROC,area under the receiver operating

cohort

cohort.


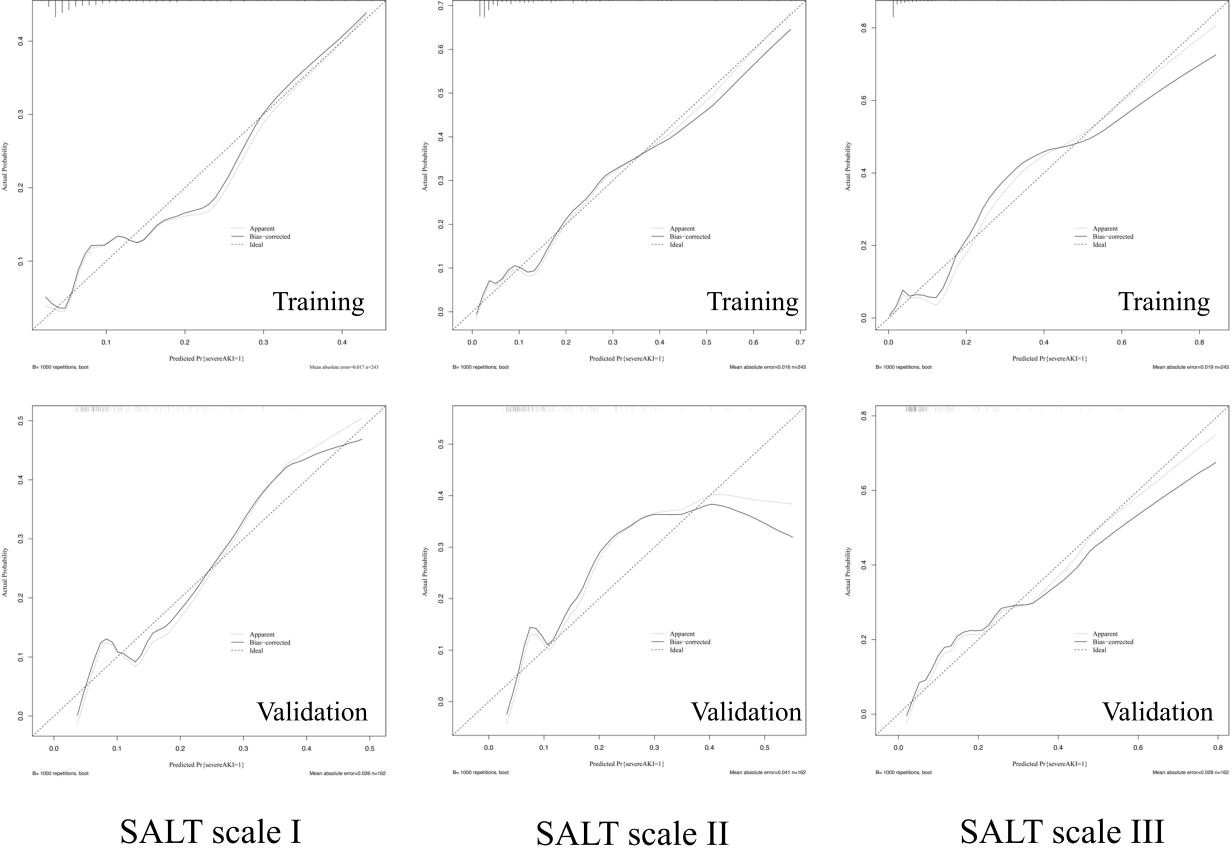


characteristic curve,ROC,receiver operating characteristic curve

Fig.S4. The calibration curves of the three nomograms in the Severe AKI post-LT (SALT) Scale for both the training and validation cohort.AKI,acute kidney injury

# Supplementary Tables:

## Table S1. Performance Comparison of Predictive Models for Severe AKI Post-LT

Training cohort Validation cohort

Model

| RO u i i PPV | | | | | NPV | RO | u i i PPV NPV | | | | |
| --- | --- | --- | --- | --- | --- | --- | --- | --- | --- | --- | --- |
| C racy tivit ficit C racy tivit ficit  y y y y  XGboos 0.91 0.83 0.87 0.82 0.40 0.97 0.78 0.79 0.80 0.69 0.31 0.93 | | | | | | | | | | | |
| t 4 | 8 | 7 | 6 | 1 | 5 | 7 | 4 | 6 | 4 | 0 | 8 |
| Logistic 0.88 | 0.81 | 0.81 | 0.82 | 0.35 | 0.96 | 0.88 | 0.80 | 0.88 | 0.75 | 0.34 | 0.96 |
| 7 | 7 | 3 | 3 | 7 | 9 | 5 | 8 | 2 | 6 | 1 | 0 |
| Random 0.99 | 0.97 | 0.97 | 0.96 | 0.96 | 0.97 | 0.72 | 0.88 | 0.59 | 0.82 | 0.15 | 0.90 |
| Forest 4 | 6 | 8 | 3 | 1 | 7 | 3 | 8 | 5 | 8 | 4 | 5 |
| Adaboo 0.95 | 0.84 | 0.93 | 0.83 | 0.41 | 0.98 | 0.82 | 0.78 | 0.79 | 0.76 | 0.29 | 0.95 |
| st 4 | 4 | 8 | 2 | 7 | 5 | 7 | 9 | 3 | 8 | 4 | 2 |
| MLP 0.57 | 0.72 | 0.44 | 0.76 | 0.14 | 0.91 | 0.53 | 0.69 | 0.56 | 0.66 | 0.08 | 0.89 |
| 9 | 9 | 7 | 8 | 1 | 5 | 4 | 3 | 6 | 0 | 1 | 8 |
| SVM 0.59 | 0.82 | 0.49 | 0.86 | 0.40 | 0.93 | 0.58 | 0.81 | 0.50 | 0.86 | 0.29 | 0.92 |
| 2 | 0 | 2 | 6 | 5 | 2 | 7 | 8 | 3 | 2 | 2 | 7 |
| KNN 0.88 | 0.87 | 0.97 | 0.69 | 0.45 | 0.93 | 0.64 | 0.82 | 0.57 | 0.70 | 0.24 | 0.90 |
| 7 | 9 | 7 | 1 | 7 | 9 | 8 | 6 | 8 | 4 | 2 | 8 |

AU Acc

Sens

Spec

AU Acc

Sens

Spec

AUROC=area under the receiver operating curve; PPV=Positive predictive value;NPV=negative predictive value; XGBoos=eXtreme Gradient Boosting ; AdBoost=Adaptive Boosting; MLP= Multilayer Perceptron ; SVM=Support Vector Machine;KNN=K-Nearest-Neighbor

Table S2.The tuned hyperparameters of employed machine learning models

Models Major parameters

XGboost L2 regularization with weight =1

learning rate =0.001

the maximum tree depth =3

Logistic Regression L2 regularization with weight =1 Maximum Number Of Iterations=100 tolerance=0.0001

RandomForest criterion= gini, maximum tree depth= 10 minimum impurity decrease= 0.0 number of estimators=20

AdaBoost learning rate= 1.0

number of estimators=50

MLP hidden layer sizes= (30, 30)

max number of iterations= 10

SVM kernel type=rbf

tolerance=0.001

KNN number of neighbors= 5

weights type= uniform

XGBoos= eXtreme Gradient Boosting; AdBoost=Adaptive Boosting; MLP=Multilayer Perceptron ;SVM=Support Vector Machine;KNN=K-Nearest-Neighbors

Table S3.Clinical characteristics of the post hoc internal training cohort(testing cohort)

Subjects Overall n=132

EBL[M(P25,P75),mL

Non Severe AKI n=116

Severe AKI n=16

1000.0

P value

]

Meld score [M (P25,

800.0(400.0-1000.0) 600.0 (400.0-1000.0)

(750.0-2000.0)

0.007

P75)]

13.0(8.5;21.9) 13.0(8.5-19.9) 16.6 (10.0-33.4) 0.024

ALT[M(P25,P75),u/l

]

R value

625.50(347.5-1097.2

)

592.50(350.5-1075.0) 846.0(296.2-1452.2)

0.458

0.008

[M(P25,P75),,min]

D-dimer[M(P25,P75)

, ug/ml]

7.40(6.2-9.9) 7.2 (6.2-9.3) 9.0(8.2;13.9)

2.3(1.2-5.5) 2.3 (1.1-4.5) 4.3(2.4-31.8)

0.006

EBL,Estimated blood loss;MELD, model for end-stage liver disease;ALT, alanine transaminase;TEG-R,thromboelastography reaction time
